# Upregulation of ECT2 Predicts Adverse Clinical Outcomes and Increases 5-Fluorouracil Resistance in Gastric Cancer Patients

**DOI:** 10.1155/2021/2102890

**Published:** 2021-07-28

**Authors:** Hua Zhang, Yuan Geng, Chunhui Sun, Jin Yu

**Affiliations:** ^1^Department of Pharmacy, Yantaishan Hospital, Yantai 264000, Shandong, China; ^2^Department of Obstetrics, Medical Insurance Office, Qingdao Municipal Hospital, Qingdao 266000, Shandong, China; ^3^Department of Hepatobiliary Surgery, The Third People's Hospital of Qingdao, Qingdao 266041, Shandong, China; ^4^Department of Oncology, Jinan Central Hospital, Cheeloo College of Medicine, Shandong University, Jinan 250013, Shandong, China

## Abstract

**Background:**

The abnormal expression and prognosis prediction of epithelial cell transforming sequence 2 (ECT2) in gastric cancer (GC) has been reported. However, the effect of ECT2 on 5-fluorouracil (5-Fu) resistance in GC is unclear. This research aims to solve the abovementioned problems.

**Methods:**

Gene expression was detected by RT-qPCR and Western blot analysis. Cell viability was evaluated by the colony formation assay, MTT assay, and flow cytometric analysis. Transwell and wound healing assays were used to detect cell metastasis.

**Results:**

Upregulation of ECT2 was found in stomach adenocarcinoma (STAD) and GC tissues. In addition, high ECT2 expression can predict adverse clinical outcomes in GC patients. More importantly, ECT2 knockdown weakened the resistance of 5-FU in GC cells. ECT2 silencing reduced the cell migratory and invasive abilities of GC cells treated with 5-FU. We also found that downregulation of ECT2 increased 5-FU sensitivity in GC cells by downregulating P-gp, MRP1, and Bcl-2.

**Conclusion:**

Upregulation of ECT2 can predict adverse clinical outcomes and increase 5-FU resistance in GC patients.

## 1. Introduction

Gastric cancer (GC) is one of the cancers with the highest incidence in the world [1]. Various gastric diseases, *Helicobacter pylori* (Hp) infection, poor diet, environment, and genetics can cause GC [2]. The incidence of GC increases significantly with age, and the peak age of onset is 50–80 years [3]. China is a high incidence area of GC. GC accounts for nearly a quarter of cancer deaths in China [4]. Moreover, the early diagnosis rate of GC is low, about 10%. Most patients with GC are in the middle and advanced stages at the time of diagnosis. The 5-year survival rate is about 7–34% [5]. Recently, more and more methods are being used to treat GC, such as radiotherapy, chemotherapy, cell membrane-derived biomimetic nanotechnology, and cancer nanomedicine [6, 7]. Preoperative chemotherapy can shrink the tumor and increase the chance of radical surgery and cure. However, the resistance of cancer cells to chemotherapy drugs can lead to chemotherapy failure [8]. Therefore, exploring new methods to weaken the drug resistance of cancer cells is of great significance to improve the success rate of chemotherapy.

Commonly used chemotherapy drugs are 5-fluorouracil (5-Fu), tegafur, mitomycin, doxorubicin, paclitaxel, cisplatin, or carboplatin [9, 10]. Many studies have shown that no-coding RNAs or genes can affect the resistance of cancer cells. For example, TRIM37 increased the resistance to CDDP in GC [11]. MiR-95-3p acted as a contributing factor for cisplatin resistance in human GC cells by targeting EMP1/PI3K/AKT signaling [12]. The exosomal miR-223 enhanced the resistance of doxorubicin in GC [13]. In addition, it was found that upregulation of KLF17 increased 5-Fu sensitivity in GC cells [14]. Here, the effect of ECT2 on 5-Fu resistance was investigated in GC.

ECT2 has been found to be involved in the development of various human cancers. Increased expression of ECT2 has been found in many malignant tumors, such as breast cancer [15], cholangiocarcinoma [16], and hepatocellular carcinoma [17]. Functionally, the ECT2/PSMD14/PTTG1 axis promoted the proliferation of gliomas by stabilizing E2F1 [18]. The inhibition of ECT2 induced by small interfering RNA suppressed the progression of osteosarcoma [19]. ECT2 overexpression also promoted the polarization of tumor-associated macrophages in hepatocellular carcinoma [20]. More importantly, upregulation of ECT2 has been found in stomach adenocarcinoma (STAD) and GC tissues [21]. However, the effect of ECT2 on 5-Fu resistance in GC cells has not been reported yet.

Here, the expression level of ECT2 was first detected in GC. The correlation between the expression of ECT2 and the clinical outcome of GC patients was also confirmed. In addition, we have also explored how ECT2 affects 5-Fu resistant in GC cells. Our research may provide a new way of thinking about weakening 5-Fu resistance.

## 2. Materials and Methods

### 2.1. Patients

Tissue samples of 66GC patients were collected from Jinan Central Hospital between July 2018 and July 2020. The content of the study is informed to everyone, and we have obtained their informed consents. All participants received only surgical treatment. Our study was approved by the Institutional Ethics Committee of Jinan Central Hospital.

### 2.2. Bioinformatics Analysis

The expression and prognosis of ECT2 in GC patients were analyzed by box plots and survival plots in the GEPIA database (http://gepia.cancer-pku.cn/).

### 2.3. Cell Culture and Transfection

Normal gastric mucosal cell lines GES-1 and AGS and NCI-N87GC cells were purchased from ATCC (Manassas, VA, USA). The above cells were cultured in RPMI-1640 medium (Gibco, USA) containing 10% FBS (37°C, 5% CO_2_). ECT2 siRNA (si-ECT2) and si-control (si-NC) were purchased from GenePharma (Shanghai, China). Lipofectamine 2000 (Invitrogen, Carlsbad, USA) was used for cell transfection according to the manufacturer's instructions.

### 2.4. RT-qPCR

The total RNA was extracted with TRIzol reagent (Thermo Fisher Scientific). PrimeScript-RT Kit (Madison, WI, USA) was used to synthesize complementary DNA (cDNA). RT-qPCR was performed using SYBR® Premix-Ex-Taq™ (Takara, TX, USA). The internal reference is GAPDH. The specific primer pairs were as follows: ECT2, sense: 5′-ACT ACT GGG AGG ACT AGC TTG-3′ and antisense: 5′-CAC TCT TGT TTC AAT CTG AGG CA-3′; GAPDH, sense: 5′-GGA GCG AGA TCC CTC CAA AAT-3′ and antisense: 5′-GGC TGT TGT CAT ACT TCT CAT GG-3′. The 2^−△△ct^ method was used to measure the relative expression of ECT2.

### 2.5. Western Blot Analysis

Protein samples were isolated by using RIPA lysis buffer (Beyotime Biotechnology). Then, the protein samples were separated by 10% SDS-PAGE. After transferring to the PVDF membrane, the protein was blocked with 5% skimmed milk. Next, the protein samples were incubated with Bax, Bcl-2, MRP1, P-gp, GST-*π*, and GAPDH primary antibodies (Abcam, Cambridge, MA, USA) at 4°C overnight. After washing 3 times, the protein samples were incubated with the corresponding secondary antibody (Abcam, USA) for 2 h. Finally, the ECL detection system (Thermo Fisher Scientific, Inc.) was used to visualize the blots. The relative level of protein expression was analyzed using ImagePro plus software (version 6.0; Media Cybernetics Inc., Rockville, MD, USA) and is represented as the density ratio versus GAPDH.

### 2.6. Cell Viability Assay

The half maximal inhibitory concentration (IC50) of AGS on 5-FU was detected by the MTT assay. AGS cells were cultured in 96-well plates for 24 h. Next, different concentrations of 5-FU were added to treat the AGS cells for 48 h. After that, MTT solution was added to incubate the cells at 37°C for 4 h. Finally, the absorbance value (OD = 490 nm) was measured.

### 2.7. Colony Formation Assay

The transfected AGS cells were cultured in 6-well plates (1000 cells/well) for three days. Then, 5-FU was added to treat the cells for 10 days. Next, the cells were stained with 0.1% crystal violet. Finally, colonies were observed by a light microscope.

### 2.8. Flow Cytometric Analysis

The transfected AGS cells were suspended in Annexin-binding buffer and harvested. Then, the cells were stained with Annexin V/FITC and PI solution (KeyGEN Biotech, Nanjing, China) and incubated in the dark for 15 min at room temperature. Finally, flow cytometry analysis was used to assess cell apoptosis.

### 2.9. Wound Healing Assay

AGS cells (1 × 10^3^ cells/well) were cultured in a 6-well plate for 24 h. After reaching 90% confluence, scratches were generated by a 200 *μ*l pipette tip. A light microscope was used to evaluate the wound width at 0 and 24 h. Images were captured by THUNDER Imagers (Leica Microsystems). Wound distance was quantified by ImageJ Software version 1.6 (National Institutes of Health, Bethesda, MD, USA).

### 2.10. Transwell Assay

Cell migration and invasion were analyzed by performing transwell assays. Transwell chambers (8 *μ*m pore size; Millipore) were applied to evaluate cell migration and invasion. For the invasion assay, AGS cells (4 × 10^3^ cells/well) were added into the upper chamber, which was precoated with Matrigel (3.9 ug/ul). For the migration assay, AGS cells (4 × 10^3^ cells/well) were added into the upper chamber, which was not precoated with Matrigel. Lower chamber was added with RPMI-1640 medium (10% FBS). Following incubation for 24 h at 37°C with 5% CO_2_, the migrated and invasive cells were stained with 0.1% crystal violet. The number of moved cells was observed under a light microscope.

### 2.11. Statistical Analysis

GraphPad Prism 6 or SPSS 21.0 is used to analyze experimental data. The results are shown as mean ± SD. The difference among groups was analyzed by using the chi-squared test or one-way ANOVA with the Bonferroni post hoc test. Significant difference means *p* < 0.05.

## 3. Results

### 3.1. Upregulation of ECT2 Predicts Adverse Clinical Outcomes in GC Patients

**Figure 1:**
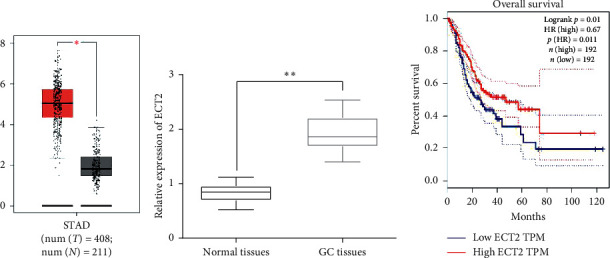
Upregulation of ECT2 predicting adverse clinical outcomes in GC patients. (a) ECT2 mRNA expression in STAD tissues (*n* = 408) and normal tissues (*n* = 211) analyzed in the GEPIA database. (b) ECT2 expression in 66GC tissues and adjacent normal tissues (*n* = 66). (c) OS rate was compared in GC patients with low or high ECT2 expression (*n* = 192). ^*∗*^*P* < 0.05. ^*∗∗*^*P* < 0.01.

First, the expression level of ECT2 was analyzed in the GEPIA database (http://gepia.cancer-pku.cn/). Compared with the control, the expression of ECT2 was increased in stomach adenocarcinoma (STAD) tissues (*p* < 0.05, Figure 1(a)). Consistently, upregulation of ECT2 in GC tissues was also found in GC samples (*p* < 0.05, Figure 1(b)). In addition, the GEPIA database showed that GC patients with high ECT2 expression had a reduced overall survival (OS) rate (*p* < 0.05, Figure 1(c)). We also found that the high expression of ECT2 was related to lymph node metastasis and TNM stage in GC patients (*p* < 0.05, Table 1). These findings imply that ECT2 may affect the development of GC.

### 3.2. Knockdown of ECT2 Enhances 5-FU Sensitivity in GC Cells

**Figure 2:**
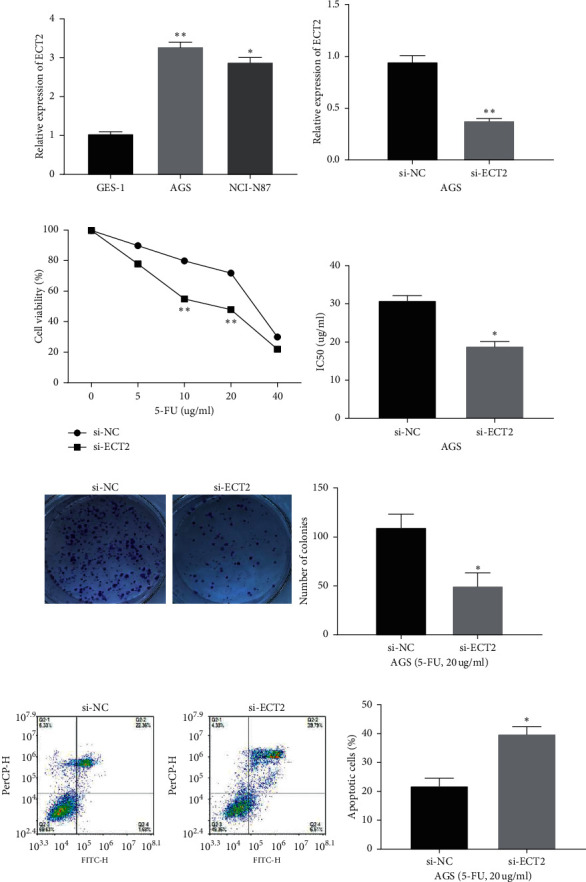
Knockdown of ECT2 enhancing 5-FU sensitivity in GC cells. (a) ECT2 expression in normal gastric mucosal cell line GES-1 and human GC cell lines AGS and NCI-N87. (b) ECT2 expression in AGS cells with si-ECT2 or si-NC. (c) Cell viability of si-NC and si-ECT2 with 5-FU treatment. (d) The IC50 value of AGS cells with si-NC and si-ECT2. (e) 5-FU sensitivity detection reconfirmed by the colony formation assay. (f) Apoptosis of AGS cells treated with 5-FU in si-NC and si-ECT2 groups. ^*∗*^*P* < 0.05. ^*∗∗*^*P* < 0.01.

Next, the expression of ECT2 in GC cells was measured. Compared with GES-1, ECT2 expression was increased in AGS and NCI-N87GC cells (*p* < 0.05, Figure 2(a)). In order to verify the effect of ECT2 on the sensitivity of 5-FU in GC cells, si-ECT2 or si-NC was transfected into AGS cells. Compared with the si-NC group, si-ECT2 reduced the expression of ECT2 in AGS cells (*p* < 0.01, Figure 2(b)). In addition, the downregulation of ECT2 enhanced the sensitivity of 5-FU in AGS cells (*p* < 0.01, Figure 2(c)). The IC50 value of the si-ECT2 group was higher than that of the si-NC group (*p* < 0.05, Figure 2(d)). At the same time, the colony formation assay also showed that knockdown of ECT2 weakened the cloning ability of AGS cells treated with 5-FU (*p* < 0.05, Figure 2(e)). The apoptosis of AGS cells treated with 5-FU was promoted by downregulation of ECT2 (*p* < 0.05, Figure 2(f)). In short, ECT2 knockdown weakens 5-FU resistance in GC cells.

### 3.3. ECT2 Silencing Inhibits the Metastasis of GC Cells Treated by 5-FU

**Figure 3:**
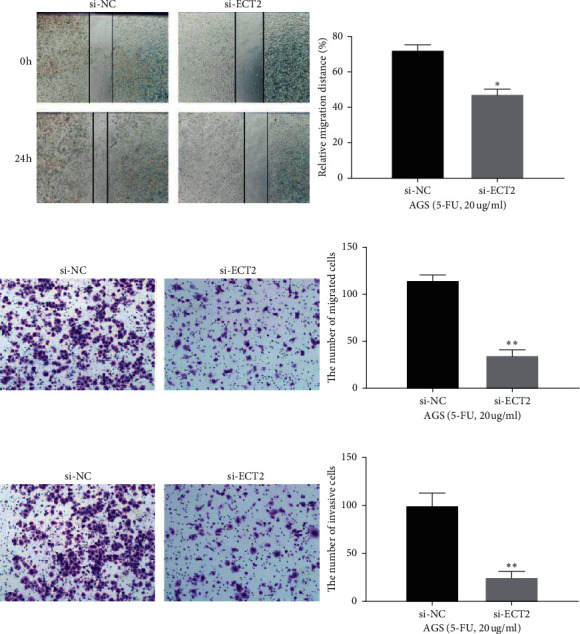
ECT2 silencing inhibiting the metastasis of GC cell lines treated by 5-FU. (a) The migration of AGS cells treated with 5-FU in si-NC and si-ECT2 groups detected by the wound healing assay. (b, c) The migration and invasion of AGS cells treated with 5-FU in si-NC and si-ECT2 groups detected by the transwell assay. ^*∗*^*P* < 0.05. ^*∗∗*^*P* < 0.01.

In order to further explore the role of ECT2 in the development of GC, the effect of ECT2 on the metastasis of 5-FU-treated AGS cell was also investigated. Both the wound healing assay and the transwell assay showed that ECT2 downregulation significantly inhibited cell migration in AGS cells treated with 5-FU (*p* < 0.05, Figures 3(a) and 3(b)). Compared with the si-NC group, cell invasion was also suppressed by ECT2 silencing in AGS cells treated with 5-FU (*p* < 0.01, Figure 3(c)). Briefly, ECT2 silencing reduced the migratory and invasive ability of 5-FU-treated GC cells.

### 3.4. ECT2 Downregulation Increases the 5-FU Sensitivity of GC Cells by Inhibiting P-gp, MRP1, and Bcl-2

**Figure 4:**
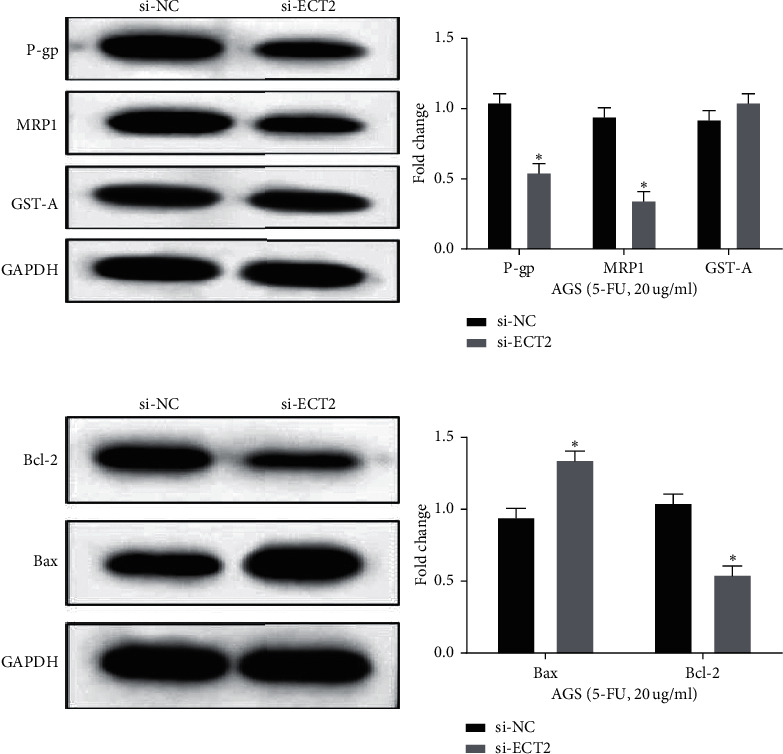
ECT2 downregulation increasing the sensitivity of GC cell lines to 5-FU by reducing the expressions of P-gp, MRP1, and Bcl-2. (a) Drug resistance-related proteins P-gp, MRP1, GST-*π* detected in AGS cells treated with 5-FU in si-NC and si-ECT2 groups. (b) Apoptosis-related proteins Bcl-2 and Bax detected in AGS cells treated with 5-FU in si-NC and si-ECT2 groups. ^*∗*^*P* < 0.05.

**Figure 5:**
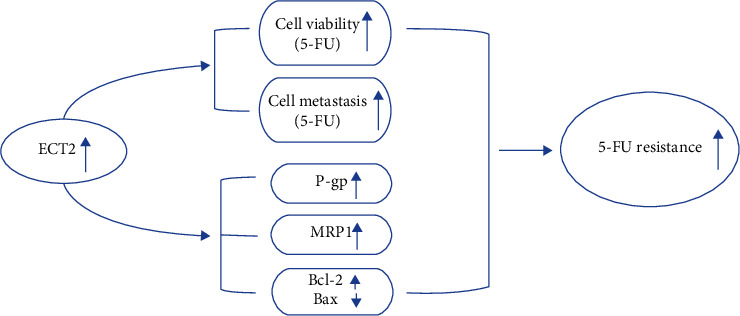
ECT2 affecting 5-FU sensitivity in GC cells by regulating cell viability, metastasis, and apoptosis-related proteins.

Finally, the effect of ECT2 on P-gp, MRP1, and GST-*π* related to drug resistance was investigated to explore the regulatory mechanism of ECT2 on 5-FU sensitivity of GC cells. We found that the expression of P-gp and MRP1 in the si-ECT2 group was significantly reduced (*p* < 0.05, Figure 4(a)). However, ECT2 has little effect on GST-*π* expression. In addition, we also explored how ECT2 regulates the apoptosis-related proteins Bcl-2 and Bax. Downregulation of ECT2 reduced Bcl-2 expression and enhanced Bax expression in AGS cells treated with 5-FU (*p* < 0.05, Figure 4(b)). Taken together, ECT2 affects the 5-FU sensitivity of GC cells by regulating cell viability, metastasis, and apoptosis-related proteins (Figure 5).

## 4. Discussion

Recently, important functions of ECT2 have been discovered in many human diseases. In addition, the effect of ECT2 on chemoresistance has also been investigated in previous studies. For example, ECT2 can regulate the growth of triple-negative breast cancer cells through the intervention of paclitaxel [22]. In this study, the effect of ECT2 on 5-FU resistance was explored in GC cells. ECT2 expression was increased in STAD and GC tissues. Previous study also proposed the upregulation of ECT2 in STAD and GC tissues [21]. In addition, high ECT2 expression can predict adverse clinical outcomes in GC patients. More importantly, ECT2 knockdown weakened the resistance of 5-FU in GC cells. ECT2 silencing reduced the migratory and invasive ability of GC cells treated with 5-FU. The above findings reveal that ECT2 may act as a tumor promoter in the progression of GC and increase 5-Fu resistance in GC patients.

Consistent with our results, Yan Chen et al. also found the upregulation and carcinogenic effects of ECT2 in GC [23]. In addition, upregulation of ECT2 also related to the poor prognosis of GC patients [24]. The same result is also found in our research. Besides that, it has been found that upregulation of ECT2 can predict adverse clinical outcomes in GC [25]. The histologic differentiation, TNM stages, and lymph node metastasis were related to ECT2 expression in GC patients [26]. This study also demonstrated the same conclusion. Functionally, upregulation of ECT2 promoted the tumor progression of renal cell carcinoma [27]. Overexpression of ECT2 also promoted the growth and metastasis of pancreatic cancer cells [28]. In this study, ECT2 silencing restrained the proliferation and metastasis of 5-FU-treated GC cells. These findings have not been found in previous studies.

In addition, we also found that downregulation of ECT2 increased the 5-FU sensitivity of GC cells by inhibiting Bcl-2, MRP1, and P-gp. P-gp can induce drug resistance by transporting drugs outside the cell [29]. MRP1 is an important gene that triggers cell resistance [30]. Apoptosis is not only related to tumor progression but also correlated with chemotherapy resistance. Among them, Bcl-2 is an antiapoptotic protein, and Bax is a proapoptotic protein [31]. Here, downregulation of ECT2 reduced the expression of Bcl-2, MRP1, and P-gp in GC cells. On the other hand, these results prove that ECT2 can increase 5-FU sensitivity in GC cells. However, our conclusion has not been verified in in vivo experiment. The function of ECT2 will be explored in vivo in the future.

## 5. Conclusion

In conclusion, upregulation of ECT2 is observed in GC, which predicts adverse clinical outcomes in GC patients. Importantly, ECT2 knockdown attenuates 5-FU resistance in GC cells. Innovatively, we found that ECT2 may affect 5-FU sensitivity in GC cells by regulating cell viability, metastasis, and apoptosis-related proteins. This study can provide a promising treatment option for GC patients.

## Figures and Tables

**Table 1:** Relationship between ECT2 expression and clinic-pathological characteristics of GC patients.

Characteristics	Cases	ECT2		*P* value
		High	Low	
Age (years)				0.083
≥55	30	18	12	
<55	36	30	6	

Gender				0.124
Male	38	28	10	
Female	28	20	8	

Tumor size (mm)				0.095
≤5.0	26	18	8	
>5.0	40	30	10	

Differentiation				0.064
Well/moderate	22	16	6	
Poor	44	32	12	

Lymph node metastasis				0.042^*∗*^
Yes	42	30	12	
No	24	18	6	

TNM stage				0.024^*∗*^
I-II	22	15	7	
III-IV	44	33	22	

## Data Availability

The datasets used to support the findings of this study are available from the corresponding author upon request.
